# 9‐Methylfascaplysin Prevents Neuroinflammation and Synaptic Damage via Cell‐Specific Inhibition of Kinases in APP/PS1 Transgenic Mice

**DOI:** 10.1111/cns.70100

**Published:** 2024-11-19

**Authors:** Jingyang Le, Chenglong Xia, Jiayi Xu, Jinhan Cai, Chenwei Hu, Yu Bai, Huiyue Chen, Wenni Rong, Yujie Jiang, Xinming Wu, Yongmei Li, Qiyao Wang, C. Benjamin Naman, Hua Wei, Jili Zhang, Hao Liu, Xiaowei Chen, Fufeng Liu, Hongze Liang, Wei Cui

**Affiliations:** ^1^ Translational Medicine Center of Pain, Emotion and Cognition, Health Science Center Ningbo University Zhejiang China; ^2^ Key Laboratory of Advanced Mass Spectrometry and Molecular Analysis of Zhejiang Province, School of Materials Science and Chemical Engineering Ningbo University Zhejiang China; ^3^ College of Food and Pharmaceutical Sciences Ningbo University Zhejiang China; ^4^ College of Biotechnology, Tianjin University of Science & Technology; Key Laboratory of Industrial Fermentation Microbiology, Ministry of Education Tianjin Key Laboratory of Industrial Microbiology Tianjin China; ^5^ School Infirmary Ningbo University Zhejiang China; ^6^ Department of Science and Conservation San Diego Botanic Garden California USA; ^7^ Ningbo College of Health Sciences Zhejiang China; ^8^ Ningbo Kangning Hospital Ningbo University Zhejiang China; ^9^ The First Affiliated Hospital of Ningbo University Zhejiang China

**Keywords:** 9‐Methylfascaplysin, Alzheimer's disease, chemical‐proteomics, GSK3β, omics‐based approaches, phosphoproteomics, ROCK2

## Abstract

**Background:**

Alzheimer's disease (AD) is a leading neurodegenerative disorder without effective treatments. The nonlinear dynamic nature of AD pathophysiology suggested that multiple pharmacological actions of anti‐AD drugs should be elucidated. 9‐Methylfascaplysin (9‐MF) was previously designed and synthesized as a novel anti‐AD candidate.

**Methods and Results:**

In this study, 9‐MF at low concentrations significantly prevented cognitive impairments with similar efficacy as donepezil in APP/PS1 transgenic mice. In addition, 9‐MF potently reduced β‐amyloid (Aβ)‐associated neuroinflammation and tau‐associated synaptic damage in vivo. 9‐MF‐regulated microglia‐specific differentially phosphorylated proteins (DPPs) were mainly enriched in neuroinflammation, while 9‐MF‐regulated neuron‐specific DPPs were enriched in synaptic regulation, as revealed by a quantitative phosphoproteomic approach. A phosphoproteome‐kinome algorithm further identified that rho‐associated coiled‐coil kinase 2 (ROCK2) and glycogen synthase kinase 3β (GSK3β) ranked high in 9‐MF‐downregulated kinase perturbations. 9‐MF possessed high affinities for ROCK2 and GSK3β, which was confirmed by in vitro kinase activity assay. The protective effects of 9‐MF were abolished by ROCK2 knockdown in Aβ‐treated BV2 microglial cells, and by GSK3β knockdown in glyceraldehyde‐treated SH‐SY5Y neuronal cells, respectively.

**Conclusions:**

All these results supported that 9‐MF produced anti‐AD effects via cell‐specific inhibition of ROCK2 and GSK3β in microglia and neurons, respectively.

## Graphical abstract

The schematic diagram showing the 9‐MF prevented long‐term cognitive impairments via cell‐specific inhibition of ROCK2 and GSK3β in APP/PS1 transgenic mice.

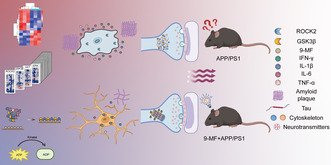

## 1 Introduction

Alzheimer's disease (AD) is the most prevalent neurodegenerative disorder worldwide [1]. The pathogenesis of AD is largely unknown, and there is still a lack of effective medications for treating this neurodegenerative disorder [2]. The hallmarks of AD are primarily senile plaques and neurofibrillary tangles, which are consisted by aggregated β‐amyloid (Aβ) and hyperphosphorylated tau protein, respectively. Aβ could be detected in the early stage of disease, leading to the over‐activation of surrounding microglia [3]. Tau protein is a main component of microtubules in the neuron. Hyper‐phosphorylated tau detaches from the microtubules, leading to neurodegeneration and synaptic damage [4, 5]. The functional association was reported between Aβ and hyper‐phosphorylated tau. Aβ alters cellular metabolism, and triggers downstream tau hyperphosphorylation, playing a key role in AD pathogenesis. Aβ fibrils induce abnormal aggregation of tau protein in the brain of P301L Tau transgenic mice [6]. In addition, Aβ aggregates induce tau phosphorylation in vitro, further supporting the association between Aβ and tau [7]. Hyperphosphorylated tau further causes the formation of intracellular neurofibrillary tangles, which interact with presynaptic vesicles, leading to synaptic failure [8].

Aβ and tau were reported to induce different protein phosphorylation profiles in microglia and neurons, respectively, suggesting that cell‐specific pathological changes of AD might be presented [9]. Kinase inhibitors have been shown effective in treating various chronic disorders, including cancer, inflammatory disorders, and neurodegenerative disorders [10]. Inhibitors of GSK3β could attenuate tau hyperphosphorylation in neurons [11]. In addition, fasudil, an inhibitor of rho‐associated coiled‐coil kinase 2 (ROCK2), potently inhibits neuroinflammation and produces significant therapeutic effects against cerebral vasospasm after subarachnoid hemorrhage, suggesting that cell‐specific inhibition of multiple kinases might be an effective avenue to treat AD [12, 13]. And, the nonlinear dynamic nature of AD pathophysiology further suggested that multiple pharmacological actions of anti‐AD candidates should be elucidated [14].

Fascaplysin is a marine alkaloid isolated from the bacteria in sponges [15]. Fascaplysin possesses a unique chemical structure that resembles ATP, indicating that fascaplysin might inhibit multiple kinases via occupying ATP‐binding sites. Fascaplysin prevented Aβ‐induced neurotoxicity in vitro [16]. We have previously designed and synthesized 9‐methylfascaplysin (9‐MF), a synthetic analog of fascaplysin with potent neuroprotection [17, 18]. However, it is unknown whether 9‐MF is safe and efficacy for AD therapy. Moreover, the detailed mechanisms underlying the anti‐AD neuroprotection of 9‐MF, especially the involvement of kinase inhibition, were not elucidated. The primary objective of this study is to investigate whether 9‐MF produced long‐term anti‐AD effects in APP/PS1 transgenic mice. Moreover, the cell‐specific kinase inhibition of 9‐MF was elucidated.

## 2 Materials and Methods

### 2.1 Chemicals

Endotoxin‐free 9‐MF was synthesized according to our previous studies [16, 17]. For the animal study, 9‐MF was dissolved in saline with 0.2% dimethyl sulfoxide (DMSO). For an in vitro study, 9‐MF was dissolved in ddH_2_O with 0.1% DMSO.

### 2.2 Transgenic Mice and Drug Administration

APP/PS1 [B6C3‐Tg (APPswe/PSEN1dE9)85Dbo/J] transgenic mice and their wild‐type (WT) littermates, as well as 5 × FAD mice [Thy‐1 promoter‐driven overexpression of K670N/M671L (Swedish), I716V (Florida), V717I (London), M146L and L286V mutations in human APP] were purchased from Hangzhou Ziyuan Co. Ltd. (Zhejiang, China). The animals were housed in an animal facility at Ningbo University, with a constant temperature of 23°C, 50% humidity, and 12:12 h light–dark cycle. All mice used were male. All the procedures were performed according to the National Institutes of Health (NIH) Guide for the Care and Use of Laboratory Animals (NIH Publications No. 80‐23, revised 1996) and were approved by the Animal Care and Use Committee of Ningbo University (SYXK‐2019‐0005).

Forty‐eight mice at 4.5 months of age were randomly assigned into six groups, namely WT, APP/PS1, APP/PS1 with donepezil (i.p., 1 mg/kg), APP/PS1 with 9‐MF at low (i.p., 0.03 mg/kg), medium (i.p., 0.1 mg/kg), and high (i.p., 0.3 mg/kg) concentrations, with eight animals in each group. Mice in WT and APP/PS1 groups were treated with saline. Drugs were given twice weekly for 5 months.

### 2.3 Behavioral Tests

All neurobehavioral assessments were conducted randomly and in a double‐blind manner. The open field tests (OFT) were used to evaluate the exploratory and locomotor activities of animals according to a previous study [19]. The experimental apparatus of OFT was a white cube opaque box with 45 cm × 45 cm × 45 cm in size. Each mouse was placed in the box with its back facing one side of the box wall and allowed to explore freely for 5 min. The total running distance and line crossing were recorded.

The nesting scoring tests were performed according to a previous study [20]. Briefly, the mice were separated into cages one night before the experiment. Six pieces of cotton were placed in each cage, and after 24 h, a 5‐point nest‐rating scale was used for scoring.

Y‐maze tests were performed according to a previous study [21]. Testing is carried out using a Y‐shape maze with three light‐colored, opaque arms orientated at 120° angles from each other. The mouse was introduced at a particular position on the maze and allowed to explore the arms freely over a short time period. An entry occurs when all four limbs of the mouse are within an arm. An alternation was defined as consecutive entries into all three arms. Then, the number of arm entries and alternations was recorded to calculate the percentage of the alternation.

The novel object recognition (NOR) test was conducted in an open‐field arena (30 × 30 × 30 cm) as described previously [22]. The task included acclimation, training, and retention over three consecutive days. On Day 1, the animals were adapted to the experimental area for 5 min without exposure to any behavioral stimulus. On Day 2, the animals explored two identical objects (white plastic cubes, 5 × 5 × 5 cm) for 5 min. On Day 3, one of the objects was replaced with a new shape and color (a gray plastic square pyramid, 5 × 5 × 7 cm), and the animals were again adapted to the area for 5 min. The field was decontaminated with 70% ethanol solution and dry cloth between the tests. The animals explored the test area by sniffing or touching the objects with their nose and/or forepaws at a distance of less than 2 cm. Sitting or turning around the objects was not considered exploratory behavior. The exploratory behavior was evaluated manually using a video camera by an observer blinded to the test conditions. Total exploration time refers to the amount of time devoted to the location of the two objects. The cognitive function was measured using a recognition index, which is the exploration time involving either of the two objects (training session) or the novel object (retention session) compared with the total exploration time.

Morris water maze (MWM) test was conducted as previously described [23]. The tests were carried out at room temperature in a 150 cm diameter circle of water poor, which was then divided into four equal quadrants. The circle platform was placed in the first quadrant except for the last day. A computer‐based video system was used to record the motion trail of mice. Animals were trained to locate and reach the platform for five consecutive days. On the sixth day, the platform was removed as a probe trial and the mice were allowed to search the platform for 90 s. All data included the time mice spent to find the platform (5 days), the time and entry of mice cost in the target quadrant (the last day), and the motion trails were collected and analyzed.

### 2.4 Immunohistochemistry (IHC) Staining

Mice were sacrificed at 9.5 months of age. Mice were anesthetized by intraperitoneal injection of 50 mg/kg sodium pentobarbital. Additionally, the brain samples were used for biochemical studies. IHC staining was performed as previously described with modifications [24]. The whole brain was extracted, fixed with 4% paraformaldehyde, and kept in 30% sucrose solution for 48 h. Brain tissue was cut into 25 μm thick slices using a freezing microtome. The primary antibody was a mixture of ionized calcium‐binding adaptor molecule 1 (Iba‐1, 1:1000, Abcam, Cambridge, UK) and Aβ (1:500, Santa Cruz Biotechnology, California, USA). The brain slices were incubated with the primary antibody for 24 h and followed by the incubation of a secondary antibody (1:200, Abclonal, Hubei, China) for 2 h. The brain slices were stained with 4′,6‐diamidino‐2‐phenylindole (DAPI, Beijing Solarbio Science & Technology Co. Ltd., Beijing, China). Sections were observed with a Confocal laser scanning microscope (LEICA TCS SP8, Hessian, Germany), and the images were analyzed by Image J.

### 2.5 Analysis of Aβ Plaques

The settings for image acquisition, including exposure time, gain, and magnification, were kept constant across all samples to ensure comparability. Images were captured in a randomized manner to avoid bias. ImageJ was used to convert images to grayscale to simplify analysis. A median filter was used to reduce noise while preserving edges. Threshold function in ImageJ was used to distinguish Aβ plaques from the background. The same thresholding parameters were applied to all images to ensure consistency. Analyze particles function was used to identify and count Aβ plaques.

### 2.6 Skeleton Analysis of Iba‐1‐Positive Microglia

A skeleton analysis method was used to quantify microglia morphology in immunofluorescent images of fixed brain tissue. The maximum intensity projection of the Iba‐1 positive channel was enhanced to visualize all microglial processes followed by noise de‐speckling to eliminate single‐pixel background fluorescence. The resulting image was converted to a binary then cell soma area was analyzed by using Image J. Analyze Skeleton plugin was applied to all skeletonized images to collect data on the number of soma areas and branch length.

### 2.7 Enzyme‐Linked Immunosorbent Assay (ELISA)

ELISA kits (Univ, Shanghai, China) were used to quantify IFN‐γ, IL‐1β, and TNF‐α in hippocampal tissue and cells. The hippocampal tissue was taken following 10 times the volume of phosphate‐buffered saline (PBS). Then, the tissues were centrifuged at 3000 rpm for 20 min and were tested by bicinchoninic acid (BCA) protein kit (Beyotime Biotechnology, Shanghai, China). The cells were taken following five times volume of trypsin and freeze and thaw for 4 times. Then, the cells were centrifuged at 3000 rpm for 20 min and were tested by BCA kit.

### 2.8 Western Blotting Analysis

Western blotting was performed by conventional techniques using 7.5% or 12.5% sodium dodecyl sulfate‐polyacrylamide gel electrophoresis (SDS‐PAGE gels, EpiZyme, Shanghai, China, PG111 and PG113). The protein was extracted from hippocampus. Brain tissues were lysed with lysis buffer, and protein concentrations were determined by BCA protein kit (Beyotime Biotechnology, Shanghai, China). SDS‐PAGE gels were performed with 150 V for 55 min. The protein was then transferred with 300 mA for 60 min. The membrane was blocked by 5% skimmed milk for 1 h. After blocking, membranes were washed by using Tris‐buffered saline and Tween 20 (TBST, 2 mM NaCl, 10 mM Tris–HCl, and 0.1% Tween‐20) three times for 5 min, followed by overnight incubation with primary antibodies against p‐ROCK2 (1:1000, AF7143, Affinity), ROCK2 (1:1000, ab71598, Abcam), p‐Akt (S473) (1:1000, 4060, Cell Signaling Technology, Massachusetts, USA), Akt (1:1000, 9272, Cell Signaling Technology), p‐ERK (1:2000, 9101, Cell Signaling Technology), ERK (1:2000, 9102, Cell Signaling Technology), p‐GSK3β(S9) (1:1000, 9336, Cell Signaling Technology), GSK3β (1:1000, 12,456, Cell Signaling Technology), CDK5 (1:1000, 2506, Cell Signaling Technology), p‐tau(S396) (1:1000, 9632, Cell Signaling Technology), p‐tau(S404) (1:1000, 20,194, Cell Signaling Technology), tau (1:1000, ab80579, Abcam), synapsin‐1 (1:1000, 5297 s, Cell Signaling Technology), brain‐derived neurotrophic factor (BDNF, 1:1000, ab108319, Abcam), PSD95 (1:1000, 3409 s, Cell Signaling Technology), β‐tubulin (1:1000, Ac008, Abclonal), and β‐actin (1:1000, Ac006, Abclonal) over night. The next day, the membranes were washed by using TBST three times for 10 min and incubated with a secondary antibody (1:3000, 7074S, and 7076S, Cell Signaling Technology) for 1 h at room temperature. Finally, the membranes were rinsed by using TBST three times and then the ECL luminescent substrate (Amersham Biosciences, Buckingham‐shire, UK) was added for color development. The analysis was performed using a fully automated chemiluminescence image analysis system (Tanon Science & Technology Co. Ltd., Shanghai, China). Data were evaluated as optical density.

### 2.9 TEM Examination

Hippocampal slices were postfixed in a fluid mixture of 2.5% glutaraldehyde, 2.0% paraformaldehyde, 0.1 M PBS for 24 h. Samples were prepared and examined using standard procedures. Ultrathin sections (80 nm) were stained with uranyl acetate and lead acetate and viewed at 80 kV under an HT‐7800 Electron Microscope. Synapses were identified by the presence of synaptic vesicles and postsynaptic densities [25]. Synapse thickness number was quantified by Image J.

### 2.10 Phosphoproteomic Approach and Bioinformatics Analysis

Polypeptide preparation was performed according to a reported protocol [9]. Each sample (5 mg) was dissolved in 0.1 mL NH_4_HCO_3_ buffer (50 mM, containing 8 M urea) and incubated with 0.2 mL DL‐dithiothreitol (0.1 M) for 30 min at 37°C. After the addition of 0.2 mL indoleacetic acid solution (0.2 M), the reaction mixture was reacted in the dark at room temperature for 30 min. The mixture was diluted to 1.0 mL NH_4_HCO_3_ buffer (50 mM) and incubated with trypsin (1:50) for 16 h at 37°C.

The preparation of phosphonate‐functionalized adsorbents and phosphorylated peptide enrichment was performed according to the methods of our previous report [26]. The polypeptides (1 mg) were dissolved in 500 μL loading buffer [50% acetonitrile containing 1% trifluoroacetic acid (TFA)] and treated with 2 mg of nanocomposite adsorbent. The dispersion was oscillated at 37°C for 30 min. The nanocomposite was separated and washed with loading buffer three times and then the captured phosphorylated peptides were eluted by 30 μL buffer (0.4 M NH_4_OH). The eluent was lyophilized to dryness and desalted with a C18 cartridge and analyzed by nanoscale liquid chromatography with tandem mass spectrometry (nano‐LC–MS/MS).

LC–MS/MS analysis was performed in accordance with our earlier studies [26]. Phosphopeptides were analyzed on an EASY‐nLC 1200 system (Thermo Scientific, Massachusetts, USA) coupled with Orbitrap Fusion Lumos mass spectrometer (Thermo Finnigan, California, USA) with nanoelectronspray ion source. Lyophilized sample was dissolved in 20 μL TFA (1%), loaded on a trap C18 column (100 μm × 2 cm, 5 μm, 100 Å, Thermo Scientific), and separated through an analytical C18 column (75 μm × 25 cm, 5 μm, 100 Å, Thermo Scientific) with mobile phase A (2% acetonitrile with 0.1% formic acid) and mobile phase B (90% acetonitrile with 0.1% formic acid) for 60 min. Elution gradient was set as follows: 0–40 min, 5%–28% B; 40–42 min, 28%–90% B; 42–60 min, 90% B. The flow rate was 250 nL/min. The mass spectrometer was run in positive mode. Full‐scan MS spectra (m/z 350–1600) were acquired using the Orbitrap with a resolution of 60,000 at m/z 200. MS^2^ spectra were obtained with a resolution of 15,000 at m/z 200.

Cell‐specific enrichment analysis and a phosphoproteome‐kinome algorithm were performed as previously described [27, 28]. DAVID Bioinformatics Resources 6.8 was used for functional enrichment analysis of Gene Ontology (GO). The Kyoto Encyclopedia of Genes and Genomes (KEGG) was evaluated using ChiPlot (https://www.chiplot/). STRING database version 10.5 (http://string‐db.org/) was used for protein–protein interaction (PPI) network analysis, and the interaction network was visualized with Cytoscape 3.9.1. Principal component analysis (PCA) and Pearson's correlations were visualized on Bioinformatics (http://www.bioinformatics.com) [9].

### 2.11 Chemical‐Proteomic Approach, Kinase Activity Assay, and Molecular Docking Analysis

A chemical‐proteomic analysis was performed as previously described [29]. And in vitro ROCK2 and GSK3β inhibitory activities were determined by ADP‐Glo kinase assay (Promega, Wisconsin, USA). The X‐ray structure of ROCK2 and GSK3β was retrieved from the RCSB Protein Data Bank (PDB ID: 6ED6 and 1Q5K) [16]. Molecular docking was carried out on Gold 5.2.2 (Cambridge Crystallographic Data Centre Software Ltd., Cambridge, U.K.). The binding pocket was defined by the residues within a 5 Å radius of 9‐MF. A genetic algorithm and Gold score were used to calculate and select the best docking conformation in the binding pocket.

### 2.12 Preparation of Aβ_1–42_ Oligomers

Aβ_1–42_ oligomers were prepared as previously reported [30]. Aβ_1–42_ powder (GL Biochem, Shanghai, China) was dissolved in hexafluoro‐isopropanol (HFIP, Sigma‐Aldrich, California, USA). 100 μL Aβ_1–42_ monomer solution was added to 900 μL Milli‐Q water. HFIP in the solution was evaporated by N_2_ blowing until the final concentration of Aβ_1–42_ monomer solution at 50 μM. Aβ_1–42_ monomer solution was mixed with or without different concentrations of phloroglucinol (12.5–100 μM), and Aβ_1–42_ concentration was at 10 μM. The samples were kept at the room temperature with constant vibrating and containing mainly Aβ_1–42_ oligomers.

### 2.13 Cell Culture and siRNA Transfection

BV2 cells and SH‐SY5Y cells were cultured in high glucose modified Eagle's medium (DMEM) which was supplemented with 10% fetal bovine serum (FBS) and penicillin (100 μ/mL)/streptomycin (100 μg/mL) with 5% CO_2_ at 37°C. The medium was refreshed every 2 days. Before experiments, the medium was changed to DMEM supplemented with 1% FBS for 24 h. 3‐(4,5‐Dimethylthiazol‐2‐yl)‐2,5diphenyltetrazolium bromide (MTT) assay and fluorescein diacetate (FDA)/propidium iodide (PI) double staining were performed to measure cytotoxicity [31]. siRNA was synthesized by GenePharma Co. Ltd. (Shanghai, China). Cells in the logarithmic growth phase were seeded into 6‐well plates 24 h prior to transfection. Cells were transfected with siRNA using Lipo8000 (Beyotime Biotechnology) according to the manufacturer's protocol.

siGSK3β:

- Sense strand: 5′‐GACGCUCCCUGUGAUUUAUTT‐3′
- Antisense strand: 5′‐AUAAAUCACAGGGAGCGUCTT‐3′

siROCK2:

- Sense strand: 5′‐GCAGCAACUUUGACGACAUTT‐3′
- Antisense strand: 5′‐AAUGUCGUCAAAGUUGCUGCTT‐3′

siNC:

Sense strand: 5′‐UUCUCCGAACGUGUCACGUTT‐3′

Antisense strand: 5′‐ACGUGACACGUUCGGAGAATT‐3′.

### 2.14 Statistical Analysis

All data were tested for normal distribution before data analysis. Data were analyzed using SPSS software version 21.0 (SPSS, Inc.), and normality of data was assessed by Kolmogorov–Smirnov test. The alpha value was established at 0.05, and *p*‐values above 0.05 were considered for normality. A nonparametric test was analyzed when data distributions were not normal. The data were displayed as means ± standard derivation (SD). One‐way analysis of variation (ANOVA) or two‐way ANOVA was utilized to decide the statistical significance, and Tukey's test was applied for post hoc multiple comparisons. *p* < 0.05 was considered to have statistical significance.

## 3 Results

### 3.1 9‐MF Prevents Cognitive Impairments of APP/PS1 Transgenic Mice at 9.5 Months of Age

**FIGURE 1:**
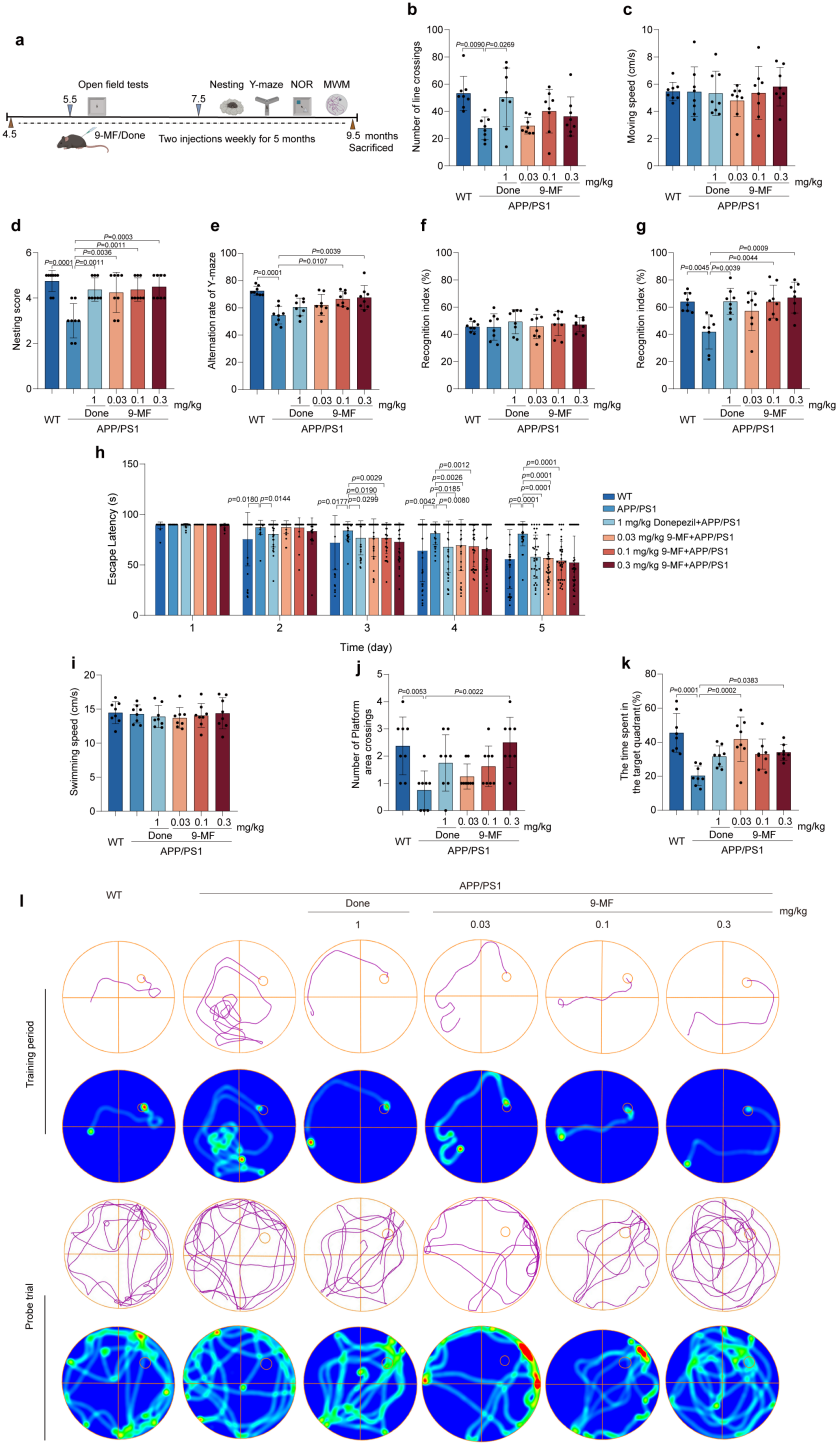
9‐MF significantly prevents cognitive impairments in APP/PS1 transgenic mice at 9.5 months of age. (a) The design of the experiment was demonstrated. In the OFT, 9‐MF did not significantly change (b) the number of line crossings or (c) the moving speed in APP/PS1 transgenic mice. (d) 9‐MF significantly improved nesting scores in APP/PS1 transgenic mice. (e) 9‐MF significantly increased the alternation rate during Y‐maze tests in APP/PS1 transgenic mice. (f) 9‐MF did not affect the recognition index during the training session of NOR tests in APP/PS1 transgenic mice. (g) 9‐MF significantly attenuated the recognition index during the retention session of NOR tests in APP/PS1 transgenic mice. (h) On the fifth day of the training period in MWM tests, 9‐MF decreased escape latency in APP/PS1 transgenic mice. During the probe trial of MWM tests, 9‐MF did not significantly alter (i) the swimming speed, but significantly increased (j) the number of platform area crossings and (k) the percentage of the time spent in the target quadrant in APP/PS1 transgenic mice. (l) Representative swimming paths of mice on the last day of the training period and the probe trial of MWM tests. The data were expressed as mean ± SD. *n* = 8. Statistical analysis was performed using One‐way ANOVA, Two‐way ANOVA, and Tukey's test.

**FIGURE 2:**
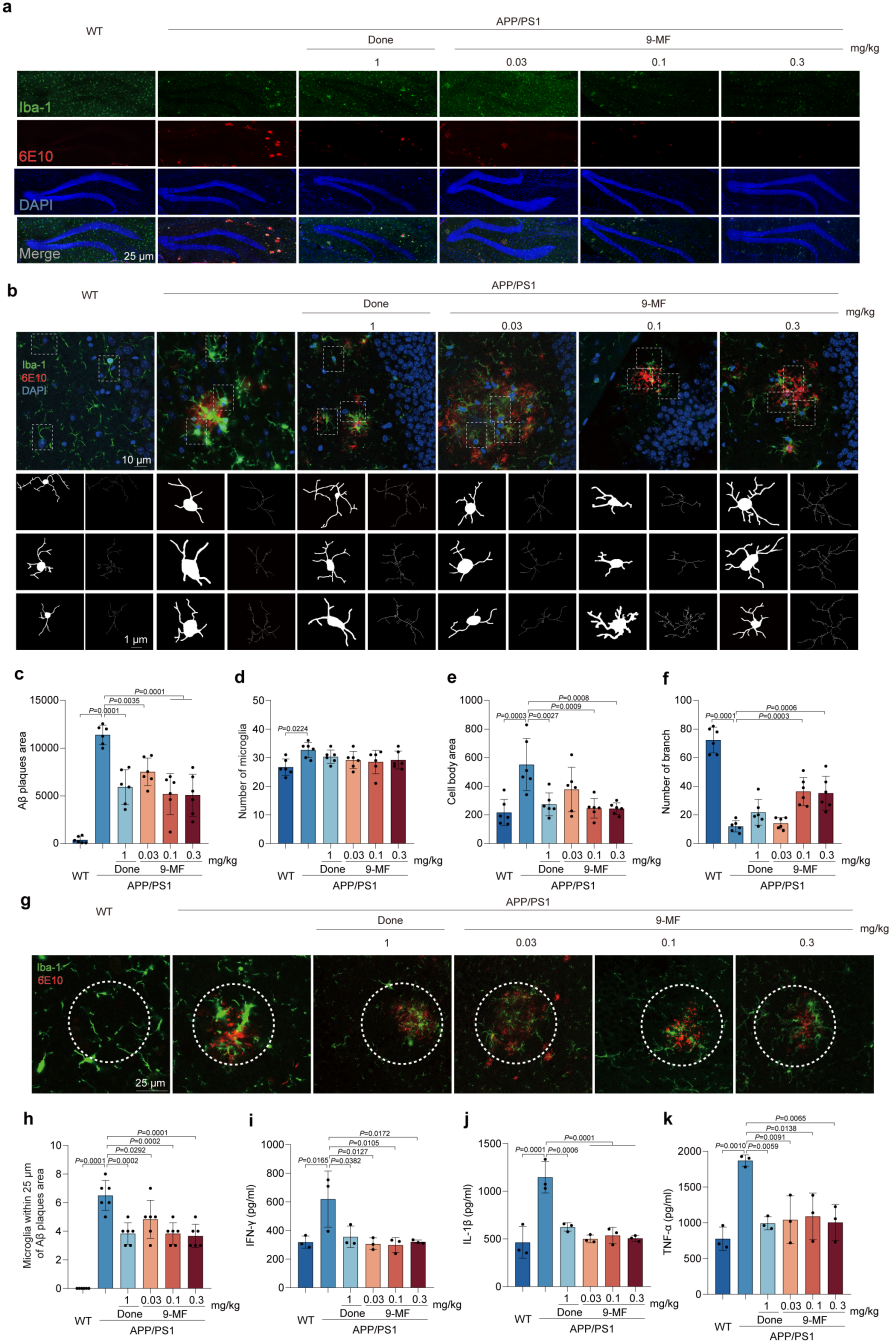
9‐ **MF** effectively reduces the amount of Aβ plaques and the overactivation of surrounding microglia in **APP** / **PS1** transgenic mice. (a) Representative immunofluorescent images of hippocampal slices stained with 6E10‐positive plaques and Iba‐1‐positive cells (20× magnification). (b) Representative immunofluorescent images of microglia and Aβ co‐localization and the cytoskeleton of microglia around Aβ plaques (60× magnification). (c) The quantification of Aβ area in (a) was shown. 9‐MF significantly prevented the increase of Aβ plaque area in APP/PS1 transgenic mice. (d) The number of microglia in (a) was shown. The quantification of (e) cell body area and (f) branches in (b) was shown. 9‐MF significantly prevented the increase of Iba‐1‐positive cell body area and the decrease of Iba‐1‐positive branches in APP/PS1 transgenic mice. (g) Representative immunofluorescence images of microglia and Aβ co‐localization (60× magnification). (h) The quantification of microglia co‐localized within 25 μm of Aβ plaque in (g) was shown. 9‐MF significantly decreased co‐localization of microglia and Aβ in APP/PS1 transgenic mice. The secretion of pro‐inflammatory cytokines was evaluated by ELISA. 9‐MF significantly decreased the secretion of (i) IFN‐γ, (j) IL‐1β, and (k) TNF‐α in the hippocampal region of APP/PS1 transgenic mice. The data were expressed as mean ± SD. *n* = 6 in (c–h), and 3 in (i–k). Statistical analysis was performed using One‐way ANOVA and Tukey's test.

The cognitive‐enhancing effects of 9‐MF were investigated in APP/PS1 transgenic mice. The arrangement of behavioral tests is demonstrated in Figure 1a. The mice were injected with 9‐MF at 0.03–0.3 mg/kg for 2 months. Body weight was measured for every 2 weeks. It was found that the body weight of mice was not significantly changed among different groups, indicating that long‐term treatment of 9‐MF at 0.03–0.3 mg/kg (*i.p*.) might be safe to animals (Extend Data Figure 1). At 5 months of age, 9‐MF did not change motor function and cognitive performance in APP/PS1 transgenic mice (Extend Data Figure 2). At 9.5 months of age, compared with WT group, the number of crossings in the OFT was decreased in APP/PS1 group (Figure 1b). However, there was no significant difference between APP/PS1 and 9‐MF + APP/PS1 groups (Figure 1b). Furthermore, the moving speed was not statistically different among groups in the OFT (Figure 1c). In nesting tests, compared with WT group, APP/PS1 groups had a decreased nesting score (Figure 1d). Moreover, 9‐MF and donepezil significantly prevented the decrease of nesting score in APP/PS1 transgenic mice (Figure 1d). In Y‐maze tests, compared with WT group, the alternation rate was significantly decreased in APP/PS1 group (Figure 1e). In addition, 9‐MF at 0.1–0.3 mg/kg increased the alternation rate compared to that in APP/PS1 group, suggesting that 9‐MF attenuated spatial cognitive dysfunction in APP/PS1 transgenic mice (Figure 1e). In the training stage of NOR tests, no detectable difference in recognition index was observed among groups (Figure 1f). In the retention stages, compared with WT group, the recognition index was significantly decreased in APP/PS1 group (Figure 1g). In addition, 9‐MF at 0.1–0.3 mg/kg, as well as donepezil at 1 mg/kg, significantly enhanced the recognition index compared with APP/PS1 group (Figure 1g). On the 5th day of training session in MWM tests, mice in APP/PS1 group spent significantly longer duration to find the platform compared to those in WT group (Figure 1h). 9‐MF at 0.3 mg/kg, significantly decreased the escape latency in APP/PS1 group, demonstrating that 9‐MF inhibited the impairments of spatial learning in mice (Figure 1h). At the same condition, the swimming speed did not statistically change among groups (Figure 1i). Compared with APP/PS1 group, WT group significantly increased the number of platform area crossings in the probe trial (Figure 1j). In addition, the number of platform area crossings was significantly increased by 9‐MF at 0.3 mg/kg (Figure 1j). Moreover, compared with APP/PS1 group, WT group significantly increased the duration in the target quadrant in the probe trial (Figure 1k). The duration in the target quadrant was significantly increased by 9‐MF at 0.3 mg/kg, as well as donepezil at 1 mg/kg in APP/PS1 transgenic mice, indicating that 9‐MF and donepezil prevented the dysfunction of spatial memory in AD animals (Figure 1k).

### 3.2 9‐MF Significantly Reduces the Amount of Aβ Plaque and Decreases the Overactivation of Surrounding Microglia in APP/PS1 Transgenic Mice

**FIGURE 3:**
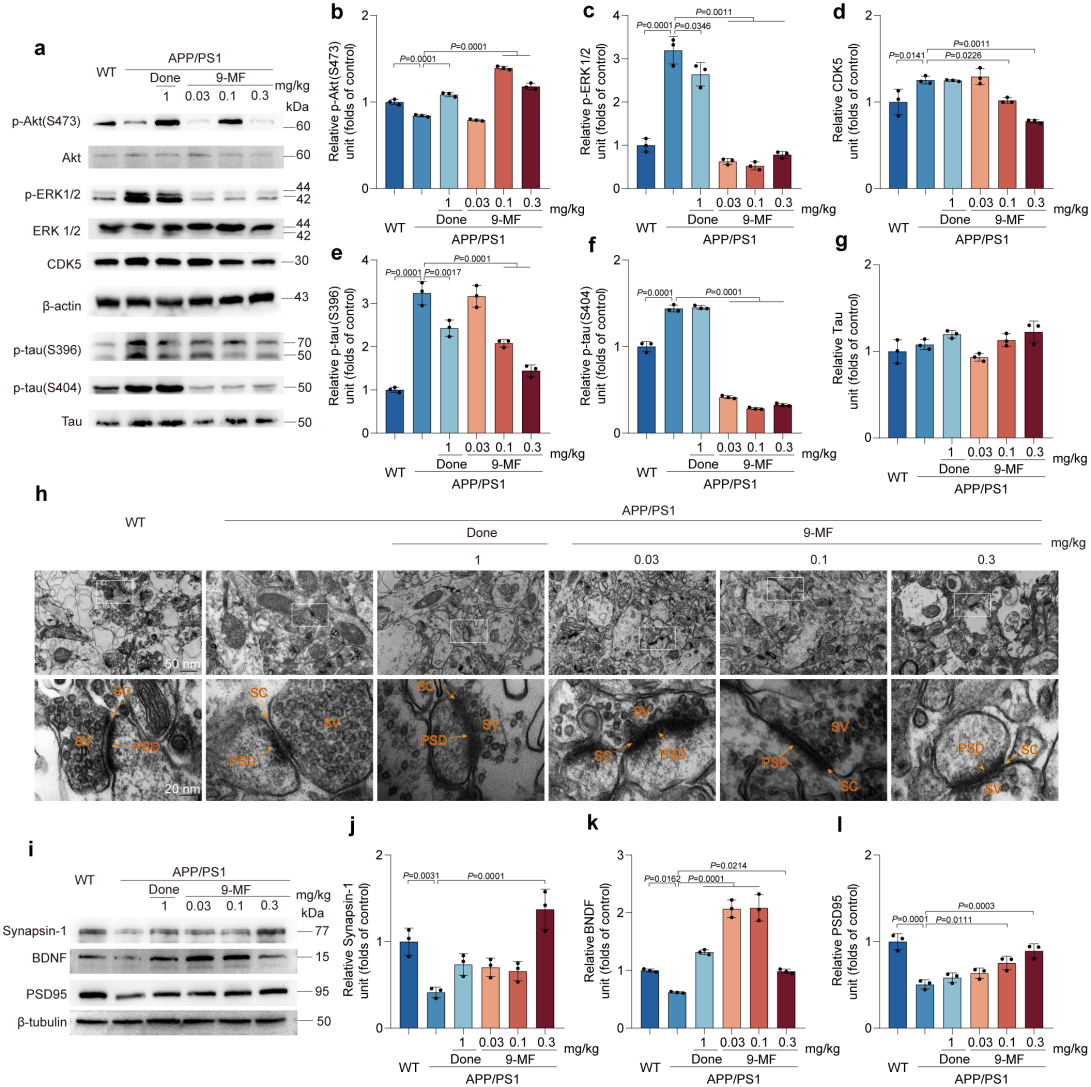
9‐MF effectively reduces tau phosphorylation and synaptic damage in the hippocampal regions of APP/PS1 transgenic mice. (a) The expressions of p‐Akt, p‐ERK1/2, CDK5, p‐tau(S396), and p‐tau(S404) were evaluated by Western blotting analysis. The quantification of (b) p‐Akt, (c) p‐ERK1/2, (d) CDK5, (e) p‐tau(S396), (f) p‐tau(S404) and (g) tau was shown. 9‐MF significantly increased the expression of p‐Akt and decreased the expression of p‐ERK1/2, CDK5, p‐tau(S396), and p‐tau(S404) in APP/PS1 transgenic mice. (h) Representative TEM images of hippocampal CA1 region. SC: Synaptic cleft; SV: Synaptic vesicles; PSD: Postsynaptic density. (i) The expressions of synapsin‐1, BDNF and PSD95 were evaluated by Western blotting analysis. The quantification of (j) synapsin‐1, (k) BDNF and (l) PSD95 was shown. 9‐MF significantly prevented the decrease of synapsin‐1, BDNF and PSD95 in APP/PS1 transgenic mice. The data were expressed as mean ± SD. *n* = 3. Statistical analysis was performed using One‐way ANOVA and Tukey's test.

6E10‐positive Aβ burden and Iba‐1‐positive microglia were evaluated in the hippocampal and cortical regions of APP/PS1 transgenic mice, respectively (Figure 2a and Extend Data Figure 3). In the hippocampus, compared with WT group, Aβ plaque area was significantly increased in APP/PS1 group (Figure 2c). The reduction in the amount of Aβ plaque was found in APP/PS1 transgenic mice treated with 9‐MF at 0.03–0.3 mg/kg and donepezil at 1 mg/kg (Figure 2c). However, 9‐MF did not significantly reduce the number of Iba‐1‐positive cells around Aβ deposits (Figure 2d). Compared with WT group, the number of microglia was increased in APP/PS1 group (Figure 2d). Moreover, compared with WT group, the cell body area was significantly increased, while the number of branches was decreased in APP/PS1 group (Figure 2e,f). And mostly, globular and rounded Iba‐1‐positive cells were discovered in the brains of APP/PS1 transgenic mice, indicating an overactivated state of microglia. In addition, the Iba‐1‐positive cells in 9‐MF at 0.1–0.3 mg/kg and donepezil at 1 mg/kg groups showed small cell body area with branches, indicating a homeostatic or resting state of microglia (Figure 2e,f). Furthermore, there was a significant difference of the number of surrounding microglia among groups (Figure 2g,h). Compared with WT group, the secretion of pro‐inflammatory cytokines, including IFN‐γ, IL‐1β, and TNF‐α, was significantly increased in APP/PS1 group, which was prevented by 9‐MF at 0.03–0.3 mg/kg, as well as donepezil at 1 mg/kg (Figure 2i–k). The similar results were found in the cortical region of APP/PS1 transgenic mice (Extend Data Figure 3). These results suggested that 9‐MF reduced the amount of Aβ plaque, and prevented Aβ‐associated neuroinflammation in APP/PS1 transgenic mice.

### 3.3 9‐MF Effectively Reduces Tau Phosphorylation and Synaptic Damage in APP/PS1 Transgenic Mice

9‐MF at 0.1–0.3 mg/kg significantly increased the expression of p‐Akt, decreased the expression of p‐ERK1/2, CDK5, p‐tau(S396), and p‐tau(S404) in the hippocampus of APP/PS1 transgenic mice (Figure 3a–g). These results suggested that 9‐MF might inhibit tau phosphorylation via inhibiting the activity of kinases responsible for tau phosphorylation. The ultrastructure of synapses in CA1 region of WT group was normal with intact cellular membranes and clearly visible synaptic cleft (Figure 3h). Compared with WT group, the postsynaptic density thickness was significantly decreased in APP/PS1 group (Table 1). And, the postsynaptic density thickness in 9‐MF at 0.03–0.3 mg/kg, as well as donepezil at 1 mg/kg groups were significantly thicker than that at APP/PS1 group (Table 1). At the same condition, the synaptic cleft width was not altered among groups (Table 1). Furthermore, compared with WT group, APP/PS1 group decreased the expressions of synapsin‐1, BDNF, and PSD95, which was significantly prevented by 9‐MF at 0.3 mg/kg (Figure 3i–l). These results suggested that 9‐MF effectively reduced tau phosphorylation and synaptic damage in APP/PS1 transgenic mice.

**TABLE 1:** Effects of 9‐MF and donepezil on thickness of postsynaptic density and width of synaptic cleft in the hippocampal region of APP/PS1 transgenic mice.

Group	Thickness of postsynaptic density (nm)	Width of synaptic cleft (nm)
WT	42.29 ± 0.87	13.47 ± 0.50
APP/PS1	35.35 ± 2.05**	14.05 ± 0.73
1 mg/kg donepezil + APP/PS1	40.58 ± 3.42*	13.93 ± 0.98
0.03 mg/kg 9‐MF + APP/PS1	43.17 ± 2.54*	13.36 ± 0.61
0.1 mg/kg 9‐MF + APP/PS1	41.66 ± 5.51*	13.46 ± 0.89
0.3 mg/kg 9‐MF + APP/PS1	41.76 ± 4.37*	13.96 ± 1.01

*Note:* Data were shown as mean ± SD, *n* = 8.

* *p* < 0.05 vs. APP/PS1 group (one‐way ANOVA and Tukey's test).

** *p* < 0.05 vs. WT group.

### 3.4 9‐MF Cell‐Specifically Inhibits the Activity of ROCK2 and GSK3β

**FIGURE 4:**
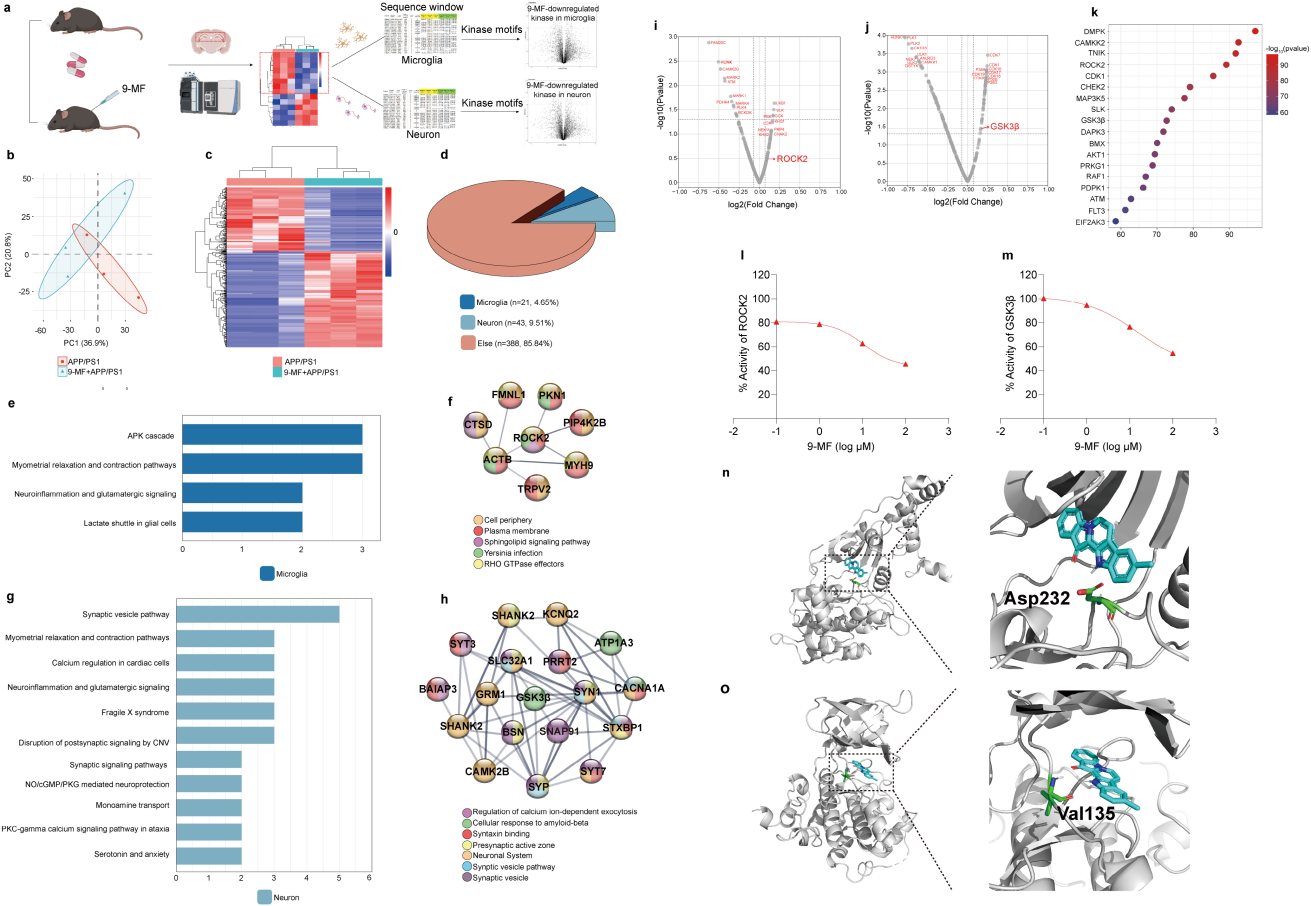
9‐MF cell‐specifically inhibits the activity of ROCK2 and GSK3β as evidenced by omics‐based approaches. (a) The experimental design of a quantitative phosphoproteomic study, cell‐specific analysis, and phosphoproteome‐kinome conversion were demonstrated. (b) A PCA plot of quantified phosphor‐peptides was shown. Samples were from APP/PS1 transgenic mice treated with or without 9‐MF. (c) DPPs between APP/PS1 and APP/PS1 + 9‐MF groups were analyzed by hierarchical clustering. (d) The percentages of neuron‐specific and microglia‐specific DPPs were demonstrated. (e) WIKI analysis of microglia‐specific DPPs. (f) A biological network was established from microglia‐specific DPPs. (g) WIKI analysis of neuron‐specific DPPs. (h) A biological network was established from neuron‐specific DPPs. (i) 9‐MF‐downregulated microglia‐specific kinase perturbations were revealed by a phosphoproteome‐kinome algorithm. (j) 9‐MF‐downregulated neuron‐specific kinase perturbations were revealed by a phosphoproteome‐kinome algorithm. (k) The interactions between 9‐MF and kinases from the hippocampal regions of 5 × FAD transgenic mice were revealed by a chemical‐proteomic approach. (l) 9‐MF directly inhibited the activity of ROCK2. (m) 9‐MF directly inhibited the activity of GSK3β. The interaction conformations between (n) 9‐MF and ROCK2, and (o) 9‐MF and GSK3β were revealed by molecular docking analysis.

The experimental design of a quantitative phosphoproteomic study, cell‐specific analysis of phosphor‐peptides, and phosphoproteome‐kinome conversion was demonstrated in Figure 4a. Mice in APP/PS1 and APP/PS1 + 9‐MF groups were sacrificed, and the phosphor‐peptides from the hippocampus were enriched. 9‐MF‐induced global changes of phosphorylated proteins were evaluated (Extend Data Figure 4). The PCA plot of quantified phosphor‐peptides in APP/PS1 and APP/PS1 + 9‐MF groups was demonstrated in Figure 4b. Totally, 470 DPPs were discovered between APP/PS1 and APP/PS1 + 9‐MF groups. The magnitude and significance of changes in phosphorylation levels were presented in a hierarchical clustering heatmap (Figure 4c), and the detailed results were demonstrated in the supplemental file. Cell‐specific DPPs were further selected. The percentages of microglia‐ and neuron‐specific DPPs were 4.65% and 9.51%, respectively (Figure 4d). WIKI pathways enrichment suggested that 9‐MF‐induced microglia‐specific DPPs were predominantly related to neuroinflammatory cascades (Figure 4e). And 9‐MF‐induced neuron‐specific DPPs were predominantly related to synaptic processes (Figure 4g). Microglia‐specific DPPs formed a ROCK2‐centralized network (Figure 4f), while neuron‐specific DPPs formed a GSK3β‐centralized network with the function of synaptic and exocytosis regulation (Figure 4h). A phosphoproteome‐kinome algorithm was used to predict 9‐MF‐downregulated cell‐specific changes of kinome. ROCK2 and GSK3β were ranked high in 9‐MF‐downregulated microglia‐ and neuron‐specific kinase perturbations, respectively (Figure 4i,j). The intensity of action between 9‐MF and proteins was evaluated in the hippocampus of 5 × FAD transgenic mice by using a chemical‐phosphoproteomic approach. 159 proteins, including 15 kinases, were found to form strong interactions against 9‐MF. The interaction intensity between 9‐MF and these 15 kinases was plotted in Figure 4k. ROCK2 and GSK3β ranked high, suggesting that 9‐MF possesses strong affinities for ROCK2 and GSK3β. In vitro kinase activity assays further demonstrated that 9‐MF directly inhibited ROCK2 and GSK3β with the IC_50_ values at 10.53 and 15.14 μM, respectively (Figure 4l,m). Molecular docking analysis demonstrated the interaction confirmation of 9‐MF with ATP‐binding sites of ROCK2 (Figure 4n) and GSK3β (Figure 4o), respectively. 9‐MF forms hydrogen binds with Val135 in GSK3β and Asp232 in ROCK2, providing a support that 9‐MF has high affinity on ROCK2 and GSK3β (Figure 4p,q). These results suggested that 9‐MF cell specifically inhibited the activity of ROCK2 and GSK3β.

### 3.5 9‐MF Exerts Anti‐Neuroinflammatory and Anti‐Neurotoxic Effects Through the Inhibition of ROCK2 and GSK3β, Respectively

**FIGURE 5:**
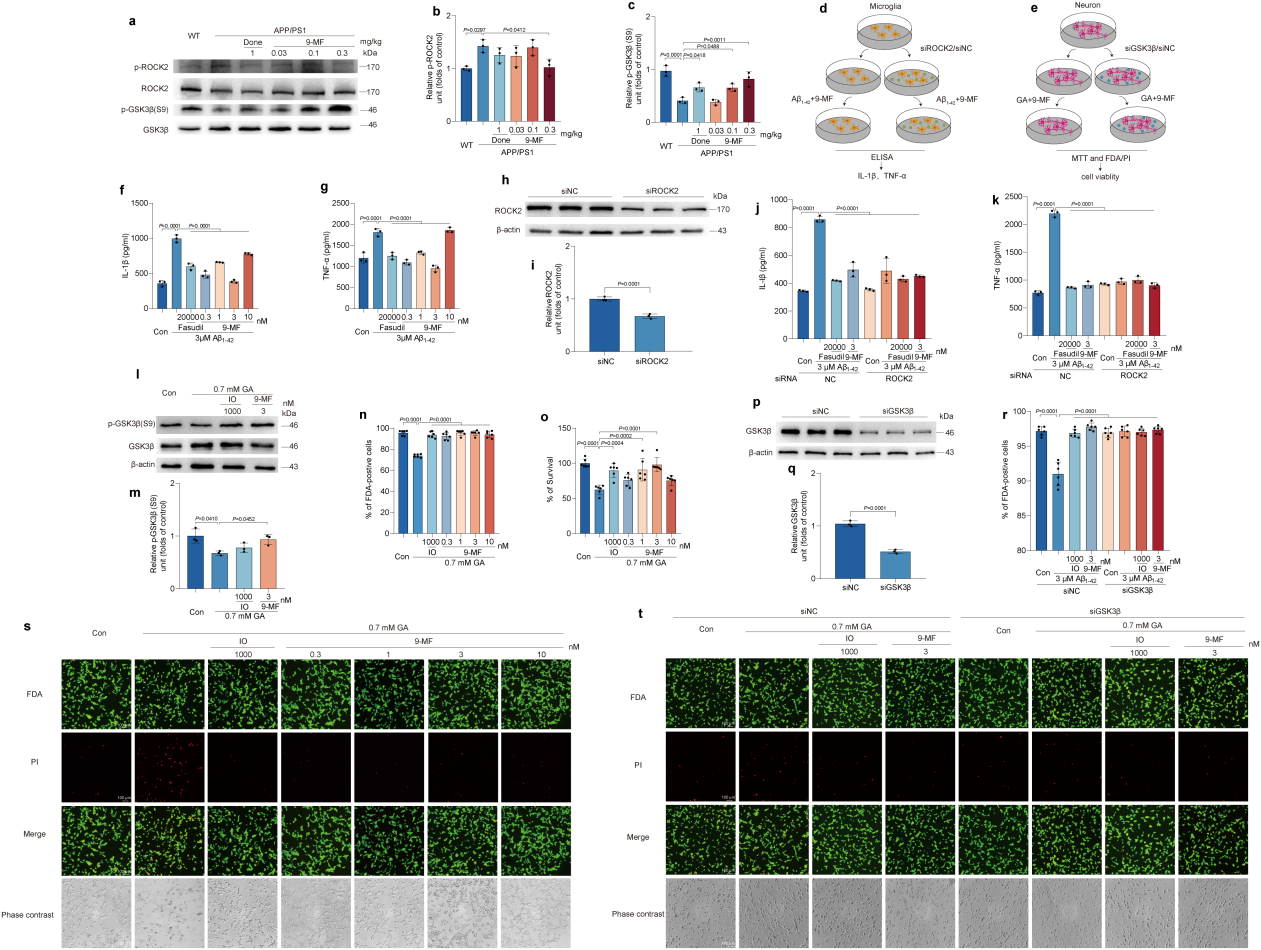
9‐MF exerts anti‐neuroinflammatory and anti‐neurotoxic effects through the inhibition of ROCK2 and GSK3β, respectively. (a) The expression of p‐ROCK2 and p‐GSK3β in the hippocampal region of APP/PS1 transgenic mice was evaluated by Western blotting analysis. The quantification of (b) p‐ROCK2 and (c) p‐GSK3β in (a) was shown. The design of experiments in (d) Aβ‐treated BV2 cells and (e) GA‐treated SH‐SY5Y cells was demonstrated, respectively. BV2 cells were treated with fasudil or 9‐MF at various concentrations for 1 h. Aβ at 3 μM was further added. After 24 h, the secretion of pro‐inflammatory cytokines was evaluated by ELISA. 9‐MF significantly prevented Aβ‐induced secretion of (f) IL‐1β and (g) TNF‐α in BV2 cells. ROCK2 was knocked down by siRNA in BV2 cells. (h) The expression of ROCK2 was evaluated by Western blotting analysis. (i) The quantification of ROCK2 expression in (h) was shown. ROCK2‐knockdown BV2 cells were treated with fasudil or 9‐MF for 2 h. Aβ at 3 μM was further added. After 24 h, the secretion of pro‐inflammatory cytokines was evaluated by ELISA. The knockdown of ROCK2 abolished the reduction of (j) IL‐1β and (k) TNF‐α by 9‐MF in Aβ‐treated BV2 cells. SH‐SY5Y cells were treated with IO or 9‐MF at various concentrations for 2 h. GA at 0.7 mM was further added. After 24 h, FDA/PI double staining and MTT assay were performed. (s) Representative FDA/PI double staining of SH‐SY5Y cells was shown. (n) FDA‐positive living cells in (s) were analyzed. (o) Cell viability was analyzed by MTT assay. 9‐MF significantly prevented GA‐induced neurotoxicity in SH‐SY5Y cells. (l) The expression of p‐GSK3β was evaluated by Western blotting analysis. (m) The quantification of p‐GSK3β expression in (l) was shown. 9‐MF significantly prevented GA‐induced decrease of p‐GSK3β expression in SH‐SY5Y cells. GSK3β was knocked down by siRNA in SH‐SY5Y cells. (p) The expression of GSK3β was evaluated by Western blotting analysis. (q) The quantification of GSK3β expression in (p) was shown. GSK3β‐knockdown SH‐SY5Y cells were treated with IO or 9‐MF for 2 h. GA at 0.7 mM was further added. After 24 h, FDA/PI double staining was performed. (t) Representative FDA/PI double staining of SH‐SY5Y cells was shown. (r) FDA‐positive living cells in (t) were analyzed. The knockdown of GSK3β abolished the neuroprotective effects of 9‐MF against GA in SH‐SY5Y cells. GA: Glyceraldehyde. The data were expressed as mean ± SD. *n* = 3 in (b, c, f–m, p, q), and 6 in (n, o, r). Statistical analysis was performed using One‐way ANOVA and Tukey's test.

The expression of p‐ROCK2 and p‐GSK3β was detected in the hippocampal region of APP/PS1 mice treated with or without 9‐MF. 9‐MF significantly prevented the increase of p‐ROCK2 and the decrease of p‐GSK3β in APP/PS1 transgenic mice (Figure 5b,c). The anti‐neuroinflammation effects of 9‐MF were further evaluated in Aβ_1–42_‐treated BV2 microglial cells (Figure 5d). Compared with control group, the secretion of IL‐1β and TNF‐α was significantly increased in Aβ_1–42_ group, which was prevented by 9‐MF at 0.3–10 nM and fasudil at 20000 nM (Figure 5f,g). ROCK2 was knocked down by using siRNA in BV2 cells (Figure 5h,i). ROCK2‐knockdown BV2 cells were treated with fasudil or 9‐MF for 2 h before the addition of Aβ_1–42_. The knockdown of ROCK2 significantly abolished the anti‐neuroinflammation effects of 9‐MF against Aβ_1–42_ in BV2 cells, suggesting that 9‐MF exerted anti‐neuroinflammation effects via the action on ROCK2 (Figure 5j,k). The neuroprotective effects of 9‐MF against glyceraldehyde (GA) were evaluated in SH‐SY5Y neuronal cells. SH‐SY5Y cells were pretreated with various concentrations of 9‐MF or IO for 2 h and then treated with 0.7 mM GA for 24 h. Cell viability in GA group was significantly lower than that of control group (Figure 5i–m). 9‐MF at 0.3–10 nM and IO at 1000 nM significantly prevented GA‐induced neurotoxicity in SH‐SY5Y cells (Figure 5n,o,s). In addition, compared with control group, GA group significantly decreased the expression of p‐GSK3β, which was prevented by 9‐MF at 3 nM or IO at 1000 nM (Figure 5l,m). GSK3β was further knocked down by using siRNA in SH‐SY5Y cells (Figure 5p,q). GSK3β‐knockdown SH‐SY5Y cells were treated with IO or 9‐MF for 2 h, followed by adding GA at 0.7 mM. After 24 h, the knockdown of GSK3β abolished the neuroprotective effects of 9‐MF against GA in SH‐SY5Y cells, suggesting that 9‐MF exerted anti‐neurotoxicity effects via the action on GSK3β in neurons (Figure 5r,t).

## 4 Discussion

In this study, we have demonstrated that 9‐MF significantly prevented cognitive impairments, Aβ‐associated microglial over‐activation, and tau‐associated synaptic damage via cell‐specific inhibition of ROCK2 and GSK3β.

APP/PS1 transgenic mice were an extensively used animal model for evaluating anti‐AD drugs [32]. These mice exhibited detectable cognitive impairment at around 8–10 months of age, reflecting the characteristics of AD as an age‐related disease. Moreover, the dysfunctions of multiple AD‐related proteins, including Aβ, tau, ROCK2, CDK5, and GSK3β, can be found in the brain of APP/PS1 transgenic mice, indicating that this animal model is suitable for studying anti‐AD drugs with the action on multiple targets [33, 34]. Therefore, we first evaluated the long‐term anti‐AD effects of 9‐MF in this model. AD mainly affects elderly, resulting in the decline of cognitive functions. It is normal that AD patients might forget to take medications. Therefore, many long‐lasting anti‐AD medications were approved. For example, food and drug administration has approved once‐weekly use of Adlarity to treat patients with AD [35]. Although we have not systematicity evaluated the pharmacokinetic/pharmacodynamic parameters of 9‐MF. 9‐MF (one time per 3 days for 10 days) could prevent scopolamine‐induced cognitive impairments in mice, suggesting that the half‐life of 9‐MF was quite long [16]. Therefore, in this study, we selected to use 9‐MF twice weekly. The drug administration was started at 4.5 months old, an age when the mice were cognitively unimpaired. After 5 months of administration, 9‐MF at 0.03–0.3 mg/kg significantly prevented cognitive impairments with the similar efficacy as that of 1 mg/kg donepezil indicating that 9‐MF is safe for long‐term usage and is effective for the prevention of AD progression. These results were consistent with our previous studies showing that 9‐MF at very low concentrations produced nonprotective effects [16, 17]. Interestingly, the 0.03 mg/kg 9‐MF group showed a longer duration in the target quadrant, as well as fewer number of platform area crossings compared to the higher dose groups in the probe trial of MWM tests. Previous studies have shown that mice could exhibit at least three searching strategies, namely spatial persistent, nonspatial concentric, and nonspatial random, in the probe trial of MWM tests [36, 37, 38]. We have reanalyzed the swimming paths of mice in the 0.03 mg/kg 9‐MF group and found that some mice exhibited nonspatial concentric strategy in the tests. However, these mice spent almost a half of duration in the target quadrant around the edge of maze and did not cross the platform area. Therefore, the results in 0.03 mg/kg 9‐MF group showed a long duration in the target quadrant, as well as a few numbers of platform area crossing in the probe trial of MWM tests.

Aβ and tau are currently considered as major neuropathological hallmarks of AD [39]. 9‐MF significantly prevented the increase of Aβ plaque area and phosphorylated tau expression in the brain of APP/PS1 transgenic, indicating that 9‐MF inhibited both Aβ‐ and tau‐associated neuropathological changes. Interestingly, many studies have suggested that Aβ mainly affects neuroinflammation in microglia while hyper‐phosphorylated tau mainly affects axonal and synaptic damage in neurons [40, 41, 42]. In our study, both neuroinflammation and synaptic damage were reversed by 9‐MF, indicating that 9‐MF might prevent Aβ‐associated biochemical changes in microglia and tau‐associated biochemical changes in neurons. In our study, a higher dose of 9‐MF (0.3 mg/kg) resulted in a relatively mild effect on BDNF expression compared to a lower dose of 9‐MF (0.1 mg/kg). Normally, a drug at high concentrations should produce greater biological effects than the same drug at low concentrations. However, BDNF might not be the direct target of 9‐MF. And, the expression of BDNF could be affected by many factors, such as transcriptional factor CREB, ion channel NMDA receptors, miR‐202‐3p and miR‐124, DNA‐binding protein MeCP2 and neurotransmitter serotonin. 9‐MF might act on the expression of BDNF via multiple targets, either inhibitory or excitatory, with different efficacies [43]. Therefore, a high dose of 9‐MF could result in a relatively mild effect on BDNF expression compared to a low dose of 9‐MF.

To further elucidate the molecular mechanisms of 9‐MF on different types of cells in the brain, a phosphoproteomic approach was performed. 21 microglia‐specific and 43 neuron‐specific DPPs were discovered between APP/PS1 and APP/PS1 + 9‐MF groups. Interestingly, neuroinflammation was enriched in microglia‐specific DPPs as a main pathway, further supporting that 9‐MF inhibited microglia‐related neuroinflammation in AD animals. At the same condition, synaptic signaling pathway was enriched in neuron‐specific DPPs as a main pathway, indicating that 9‐MF might also affect tau‐related synaptic damage in neurons.

How could 9‐MF produce anti‐AD effects via regulating cell‐specific changes of phosphorylation? Many kinases, including ROCK2 and GSK3β, were reported to be involved in Aβ‐ and tau‐associated biochemical changes [44, 45]. Importantly, fascaplysin, a precursor of 9‐MF, was reported to occupy the ATP‐binding site of kinase, suggesting that 9‐MF might inhibit kinases via acting on the ATP‐binding site [18]. Therefore, we speculated that 9‐MF inhibited AD‐related kinases to produce anti‐neuroinflammation and neuroprotective effects in different cell types.

To further identify cell‐specific perturbation of kinases by 9‐MF, a phosphoproteome‐kinome algorithm was used to computationally analyze 9‐MF‐downregulated kinases. In our study, ROCK2 and GSK3β ranked high in 9‐MF‐downregulated microglia‐ and neuron‐specific kinase perturbations, respectively, suggesting that 9‐MF differently inhibited ROCK2 and GSK3β in microglia and in neurons. We further applied a chemical‐proteomic approach and discovered the potent interactions between 9‐MF and ROCK2, as well as 9‐MF and GSK3β in the hippocampal extracts from 5 × FAD transgenic mice. Most importantly, the inhibition of kinase activity by 9‐MF was confirmed by in vitro kinase activity assays. Molecular docking analysis further suggested the interaction conformations between 9‐MF and ATP‐binding sites of ROCK2 and GSK3β. These results not only suggested that 9‐MF cell‐specially inhibited ROCK2 and GSK3β but also presented an example of how anti‐AD targets for a certain drug candidate were identified.

To prove that 9‐MF prevented AD‐associated neuroinflammation and neurotoxicity via inhibiting ROCK2 and GSK3β, Aβ‐treated BV2 cells and GA‐treated SH‐SY5Y cells were used as in vitro models of AD. The anti‐neuroinflammation effects of 9‐MF and fasudil, a reported ROCK2 inhibitor, were abolished by the knockdown of ROCK2 in BV2 cells, providing a support that 9‐MF produced anti‐neuroinflammation effects at least partially via the inhibition of ROCK2. GA was reported to induce neurotoxicity via the activation of GSK3β [46]. 9‐MF and IO, a reported GSK3β inhibitor, inhibited GA‐induced neuronal death and GSK3β activation in SH‐SY5Y cells. In addition, siRNA‐mediated knockdown of GSK3β abolished the neuroprotection of 9‐MF, suggesting that 9‐MF produced neuroprotective effects at least partially via the inhibition of GSK3β.

There are still some limitations of our study. Female animals have not been used in the study. It was reported that estrogen and luteinizing hormone in female mice could largely affect their menstrual cycle, impacting cognitive, locomotion, and behavioral performance [47]. In addition, some neurotrophins, such as BDNF, could be affected by estrogen and luteinizing hormone across the menstrual cycle, resulting in the alteration of synaptic transmission and hippocampal function [48]. Therefore, only male mice were used in the current study. Another limitation is that APP/PS1 transgenic mice are not the ideal AD model for investigating tau‐associated AD pathologies. Therefore, it is hard to elucidate the underlying mechanisms of 9‐MF on the inhibition of tau‐related neurotoxicity in this model. We plan to use other AD models, such as 3 × Tg‐AD mice, which exhibit both Aβ and tau pathologies, and to evaluate the effects of 9‐MF against tau‐related neurotoxicity in these models.

In conclusion, we have demonstrated that 9‐MF significantly prevented cognitive impairments, Aβ‐associated microglial over‐activation, and tau‐associated synaptic damage via cell‐specific inhibition of ROCK2 and GSK3β in APP/PS1 transgenic mice, providing an example of how multiple anti‐AD targets for a certain candidate were identified.

## Author Contributions

J.Y.L., C.L.X., and J.Y.X.: methodology. J.Y.L., C.L.X., J.Y.X., J.H.C., C.W.H., Y.B., and H.Y.C.: investigation. J.Y.L., C.L.X., J.Y.X., J.H.C., C.W.H., Y.B., W.N.R., Y.J.J., X.M.W., and Q.Y.W.: visualization. H.Z.L., W.C., H.L., and Y.M.L.: funding acquisition. J.Y.L., C.L.X., H.L., X.W.C., and F.F.L.: project administration. J.Y.L., C.L.X., H.W., J.L.Z., and Y.M.L.: supervision. J.Y.L., H.Z.L., W.C., and C.B.N.: writing – original draft. J.Y.L., H.Z.L., and W.C.: writing – review and editing.

## Conflicts of Interest

The authors declare no conflicts of interest.

## Supporting information


Data S1.

## Data Availability

All data are available in the main text or the supplementary information.

## References

[1] S. Zhang , F. Cao , W. Li , and N. Abumaria , “TRPM7 Kinase Activity Induces Amyloid‐β Degradation to Reverse Synaptic and Cognitive Deficits in Mouse Models of Alzheimer's Disease,” Science Signaling 16, no. 793 (2023): eade6325. 37433006 10.1126/scisignal.ade6325

[2] L. Wang , Y. L. Yin , X. Z. Liu , et al., “Current Understanding of Metal Ions in the Pathogenesis of Alzheimer's Disease,” Translational Neurodegeneration 9 (2020): 10. 32266063 10.1186/s40035-020-00189-z PMC7119290

[3] M. L. Chen , C. G. Hong , T. Yue , et al., “Inhibition of miR‐331‐3p and miR‐9‐5p Ameliorates Alzheimer's Disease by Enhancing Autophagy,” Theranostics 11, no. 5 (2021): 2395–2409. 33500732 10.7150/thno.47408 PMC7797673

[4] R. Shen , X. Zhao , L. He , et al., “Upregulation of RIN3 Induces Endosomal Dysfunction in Alzheimer's Disease,” Translational Neurodegeneration 9, no. 1 (2020): 26. 32552912 10.1186/s40035-020-00206-1 PMC7301499

[5] N. Franzmeier , A. Dewenter , L. Frontzkowski , et al., “Patient‐Centered Connectivity‐Based Prediction of Tau Pathology Spread in Alzheimer's Disease,” Science Advances 6, no. 48 (2020): eabd1327. 33246962 10.1126/sciadv.abd1327 PMC7695466

[6] J. Götz , F. Chen , J. van Dorpe , and R. M. Nitsch , “Formation of Neurofibrillary Tangles in P301l Tau Transgenic Mice Induced by Abeta 42 Fibrils,” Science (New York, N.Y.) 293, no. 5534 (2001): 1491–1495. 11520988 10.1126/science.1062097

[7] J. Busciglio , A. Lorenzo , J. Yeh , and B. A. Yankner , “Beta‐Amyloid Fibrils Induce Tau Phosphorylation and Loss of Microtubule Binding,” Neuron 14, no. 4 (1995): 879–888. 7718249 10.1016/0896-6273(95)90232-5

[8] L. Zhou , J. McInnes , K. Wierda , et al., “Tau Association With Synaptic Vesicles Causes Presynaptic Dysfunction,” Nature Communications 8 (2017): 15295. 10.1038/ncomms15295 PMC5437271 28492240

[9] Q. Wang , C. Xia , A. Zhu , et al., “Discrepancy of Synaptic and Microtubular Protein Phosphorylation in the Hippocampus of APP/PS1 and MAPT×P301S Transgenic Mice at the Early Stage of Alzheimer's Disease,” Metabolic Brain Disease 38, no. 6 (2023): 1983–1997. 37160613 10.1007/s11011-023-01209-3

[10] J. Y. Kim , Y. Bai , L. A. Jayne , et al., “A Kinome‐Wide Screen Identifies a CDKL5‐SOX9 Regulatory Axis in Epithelial Cell Death and Kidney Injury,” Nature Communications 11, no. 1 (2020): 1924. 10.1038/s41467-020-15638-6 PMC7174303 32317630

[11] K. Iijima‐Ando , L. Zhao , A. Gatt , C. Shenton , and K. Iijima , “A DNA Damage‐Activated Checkpoint Kinase Phosphorylates Tau and Enhances Tau‐Induced Neurodegeneration,” Human Molecular Genetics 19, no. 10 (2010): 1930–1938. 20159774 10.1093/hmg/ddq068 PMC2860892

[12] J. Z. Yu , C. Chen , Q. Zhang , et al., “Changes of Synapses in Experimental Autoimmune Encephalomyelitis by Using Fasudil,” Wound Repair and Regeneration: Official Publication of the Wound Healing Society [and] the European Tissue Repair Society 24, no. 2 (2016): 317–327. 10.1111/wrr.12407 26789651

[13] Y. Feng , P. V. LoGrasso , O. Defert , and R. Li , “Rho Kinase (ROCK) Inhibitors and Their Therapeutic Potential,” Journal of Medicinal Chemistry 59, no. 6 (2016): 2269–2300. 26486225 10.1021/acs.jmedchem.5b00683

[14] H. Hampel , A. Vergallo , L. F. Aguilar , et al., “Precision Pharmacology for Alzheimer's Disease,” Pharmacological Research 130 (2018): 331–365. 29458203 10.1016/j.phrs.2018.02.014 PMC8505114

[15] S. Naveen kumar , G. Rajivgandhi , G. Ramachandran , and N. Manoharan , “A Marine Sponge Fascaplysinopsis Sp. Derived Alkaloid Fascaplysin Inhibits the HepG2 Hepatocellular Carcinoma Cell,” Frontiers in Laboratory Medicine 2, no. 2 (2018): 41–48.

[16] H. Pan , H. Qiu , K. Zhang , et al., “Fascaplysin Derivatives Are Potent Multitarget Agents Against Alzheimer's Disease: In Vitro and In Vivo Evidence,” ACS Chemical Neuroscience 10, no. 11 (2019): 4741–4756. 31639294 10.1021/acschemneuro.9b00503

[17] Q. Sun , F. Liu , J. Sang , et al., “9‐Methylfascaplysin Is a More Potent Aβ Aggregation Inhibitor Than the Marine‐Derived Alkaloid, Fascaplysin, and Produces Nanomolar Neuroprotective Effects in SH‐SY5Y Cells,” Marine Drugs 17, no. 2 (2019): 121. 30781608 10.3390/md17020121 PMC6409607

[18] D. Zhang , Y. Feng , H. Pan , et al., “9‐Methylfascaplysin Exerts Anti‐Ischemic Stroke Neuroprotective Effects via the Inhibition of Neuroinflammation and Oxidative Stress in Rats,” International Immunopharmacology 97 (2021): 107656. 33895476 10.1016/j.intimp.2021.107656

[19] A. W. Schaler , A. M. Runyan , C. L. Clelland , et al., “PAC1 Receptor‐Mediated Clearance of Tau in Postsynaptic Compartments Attenuates Tau Pathology in Mouse Brain,” Science Translational Medicine 13, no. 595 (2021): eaba7394. 34039738 10.1126/scitranslmed.aba7394 PMC8988215

[20] R. Dutta , M. M. Lunzer , J. L. Auger , E. Akgün , P. S. Portoghese , and B. A. Binstadt , “A Bivalent Compound Targeting CCR5 and the Mu Opioid Receptor Treats Inflammatory Arthritis Pain in Mice Without Inducing Pharmacologic Tolerance,” Arthritis Research & Therapy 20, no. 1 (2018): 154. 30053832 10.1186/s13075-018-1661-5 PMC6062996

[21] A. K. Kraeuter , P. C. Guest , and Z. Sarnyai , “The Y‐Maze for Assessment of Spatial Working and Reference Memory in Mice,” Methods in Molecular Biology (Clifton, NJ) 1916 (2019): 105–111. 10.1007/978-1-4939-8994-2_10 30535688

[22] M. Koronyo‐Hamaoui , J. Sheyn , E. Y. Hayden , et al., “Peripherally Derived Angiotensin Converting Enzyme‐Enhanced Macrophages Alleviate Alzheimer‐Related Disease,” Brain: A Journal of Neurology 143, no. 1 (2020): 336–358. 31794021 10.1093/brain/awz364 PMC6935752

[23] E. Kelly , F. Meng , H. Fujita , et al., “Regulation of Autism‐Relevant Behaviors by Cerebellar‐Prefrontal Cortical Circuits,” Nature Neuroscience 23, no. 9 (2020): 1102–1110. 32661395 10.1038/s41593-020-0665-z PMC7483861

[24] Y. Zhou , X. Wu , L. Ye , et al., “Edaravone at High Concentrations Attenuates Cognitive Dysfunctions Induced by Abdominal Surgery Under General Anesthesia in Aged Mice,” Metabolic Brain Disease 35, no. 2 (2020): 373–383. 31916204 10.1007/s11011-019-00532-y

[25] H. Yu , L. Ma , D. Liu , et al., “Involvement of NMDAR/PSD‐95/nNOS‐NO‐cGMP Pathway in Embryonic Exposure to BPA Induced Learning and Memory Dysfunction of Rats,” Environmental Pollution (Barking, Essex: 1987) 266, no. Pt 1 (2020): 115055. 32629208 10.1016/j.envpol.2020.115055

[26] C. Xia , Q. Wang , W. Liang , et al., “Superhydrophilic Nanocomposite Adsorbents Modified via Nitrogen‐Rich Phosphonate‐Functionalized Ionic Liquid Linkers: Enhanced Phosphopeptide Enrichment and Phosphoproteome Analysis of Tau Phosphorylation in the Hippocampal Lysate of Alzheimer's Transgenic Mice,” Journal of Materials Chemistry B 10, no. 39 (2022): 7967–7978. 36124862 10.1039/d2tb01508k

[27] N. Morshed , W. T. Ralvenius , A. Nott , et al., “Phosphoproteomics Identifies Microglial Siglec‐F Inflammatory Response During Neurodegeneration,” Molecular Systems Biology 16, no. 12 (2020): e9819. 33289969 10.15252/msb.20209819 PMC7722784

[28] J. L. Johnson , T. M. Yaron , E. M. Huntsman , et al., “An Atlas of Substrate Specificities for the Human Serine/Threonine Kinome,” Nature 613, no. 7945 (2023): 759–766. 36631611 10.1038/s41586-022-05575-3 PMC9876800

[29] J. Zhang , J. Chen , K. Lv , et al., “Myrislignan Induces Redox Imbalance and Activates Autophagy in Toxoplasma Gondii,” Frontiers in Cellular and Infection Microbiology 11 (2021): 730222. 34540720 10.3389/fcimb.2021.730222 PMC8447958

[30] H. Chunhui , X. Dilin , Z. Ke , et al., “A11‐Positive β‐Amyloid Oligomer Preparation and Assessment Using Dot Blotting Analysis,” Journal of Visualized Experiments: JoVE 135 (2018): 57592. 10.3791/57592 PMC6101355 29889206

[31] Y. Xie , J. Lu , T. Yang , et al., “Phloroglucinol, a Clinical‐Used Antispasmodic, Inhibits Amyloid Aggregation and Degrades the Pre‐Formed Amyloid Proteins,” International Journal of Biological Macromolecules 213 (2022): 675–689. 35667457 10.1016/j.ijbiomac.2022.06.008

[32] B. V. Lananna , C. A. McKee , M. W. King , et al., “Chi3l1/YKL‐40 Is Controlled by the Astrocyte Circadian Clock and Regulates Neuroinflammation and Alzheimer's Disease Pathogenesis,” Science Translational Medicine 12, no. 574 (2020): eaax3519. 33328329 10.1126/scitranslmed.aax3519 PMC7808313

[33] Y. B. Hu , R. J. Ren , Y. F. Zhang , et al., “Rho‐Associated Coiled‐Coil Kinase 1 Activation Mediates Amyloid Precursor Protein Site‐Specific Ser655 Phosphorylation and Triggers Amyloid Pathology,” Aging Cell 18, no. 5 (2019): e13001. 31287605 10.1111/acel.13001 PMC6718535

[34] É. Halász , M. A. Zarbin , A. L. Davidow , L. J. Frishman , P. Gombkoto , and E. Townes‐Anderson , “ROCK Inhibition Reduces Morphological and Functional Damage to Rod Synapses After Retinal Injury,” Scientific Reports 11, no. 1 (2021): 692. 33436892 10.1038/s41598-020-80267-4 PMC7804129

[35] M. N. Sabbagh , P. Mathew , and A. Blau , “A Randomized Double‐Blind Study to Assess the Skin Irritation and Sensitization Potential of a Once‐Weekly Donepezil Transdermal Delivery System in Healthy Volunteers,” Alzheimer Disease and Associated Disorders 37, no. 4 (2023): 290–295. 37695107 10.1097/WAD.0000000000000578 PMC10664792

[36] S. Dalm , J. Grootendorst , E. R. de Kloet , and M. S. Oitzl , “Quantification of Swim Patterns in the Morris Water Maze,” Behavior Research Methods, Instruments, & Computers: A Journal of the Psychonomic Society, Inc 32, no. 1 (2000): 134–139. 10.3758/bf03200795 10758671

[37] V. Korz , “Water Maze Swim Path Analysis Based on Tracking Coordinates,” Behavior Research Methods 38, no. 3 (2006): 522–528. 17186763 10.3758/bf03192807

[38] A. Vouros , T. V. Gehring , K. Szydlowska , et al., “A Generalised Framework for Detailed Classification of Swimming Paths Inside the Morris Water Maze,” Scientific Reports 8, no. 1 (2018): 15089. 30305680 10.1038/s41598-018-33456-1 PMC6180070

[39] B. Zhou , J. G. Lu , A. Siddu , M. Wernig , and T. C. Südhof , “Synaptogenic Effect of APP‐Swedish Mutation in Familial Alzheimer's Disease,” Science Translational Medicine 14, no. 667 (2022): eabn9380. 36260691 10.1126/scitranslmed.abn9380 PMC9894682

[40] C. S. McAlpine , J. Park , A. Griciuc , et al., “Astrocytic Interleukin‐3 Programs Microglia and Limits Alzheimer's Disease,” Nature 595, no. 7869 (2021): 701–706. 34262178 10.1038/s41586-021-03734-6 PMC8934148

[41] G. Ayalon , S. H. Lee , O. Adolfsson , et al., “Antibody Semorinemab Reduces Tau Pathology in a Transgenic Mouse Model and Engages Tau in Patients With Alzheimer's Disease,” Science Translational Medicine 13, no. 593 (2021): eabb2639. 33980574 10.1126/scitranslmed.abb2639

[42] M. Xiong , H. Jiang , J. R. Serrano , et al., “APOE Immunotherapy Reduces Cerebral Amyloid Angiopathy and Amyloid Plaques While Improving Cerebrovascular Function,” Science Translational Medicine 13, no. 581 (2021): eabd7522. 33597265 10.1126/scitranslmed.abd7522 PMC8128342

[43] J. C. Arévalo and R. Deogracias , “Mechanisms Controlling the Expression and Secretion of BDNF,” Biomolecules 13, no. 5 (2023): 789. 37238659 10.3390/biom13050789 PMC10216319

[44] K. Zheng , F. Hu , Y. Zhou , et al., “miR‐135a‐5p Mediates Memory and Synaptic Impairments via the Rock2/Adducin1 Signaling Pathway in a Mouse Model of Alzheimer's Disease,” Nature Communications 12, no. 1 (2021): 1903. 10.1038/s41467-021-22196-y PMC7998005 33771994

[45] K. M. Tran , S. Kawauchi , E. A. Kramár , et al., “A Trem2R47H Mouse Model Without Cryptic Splicing Drives Age‐ and Disease‐Dependent Tissue Damage and Synaptic Loss in Response to Plaques,” Molecular Neurodegeneration 18, no. 1 (2023): 12. 36803190 10.1186/s13024-023-00598-4 PMC9938579

[46] H. Yao , G. Uras , P. Zhang , et al., “Discovery of Novel Tacrine‐Pyrimidone Hybrids as Potent Dual AChE/GSK‐3 Inhibitors for the Treatment of Alzheimer's Disease,” Journal of Medicinal Chemistry 64, no. 11 (2021): 7483–7506. 34024109 10.1021/acs.jmedchem.1c00160

[47] K. M. Frick and J. Kim , “Mechanisms Underlying the Rapid Effects of Estradiol and Progesterone on Hippocampal Memory Consolidation in Female Rodents,” Hormones and Behavior 104 (2018): 100–110. 29727606 10.1016/j.yhbeh.2018.04.013 PMC6226372

[48] R. B. Gibbs , “Levels of trkA and BDNF mRNA, but Not NGF mRNA, Fluctuate Across the Estrous Cycle and Increase in Response to Acute Hormone Replacement,” Brain Research 787, no. 2 (1998): 259–268. 9518642 10.1016/s0006-8993(97)01511-4

