# Supplementary material for: 9‐Methylfascaplysin Prevents Neuroinflammation and Synaptic Damage via Cell‐Specific Inhibition of Kinases in APP/PS1 Transgenic Mice
Source: CNS Neurosci Ther. 2024 Nov 19;30(11):e70100. doi: 10.1111/cns.70100 (PMC11576489; doi:10.1111/cns.70100)
Supplement: Supplementary file 1 — Data S1. [file CNS-30-e70100-s001.docx]

Supplementary Materials for

9-Methylfascaplysin prevents neuroinflammation and synaptic damage via cell-specific inhibition of kinases in APP/PS1 transgenic mice

Jingyang Le^a^, et al

*Corresponding author. Email: [lianghongze@nbu.edu.cn](mailto:lianghongze@nbu.edu.cn) and [cuiwei@nbu.edu.cn](mailto:cuiwei@nbu.edu.cn).

**Extended Data Figure 1. 9-MF did not significantly change the body weight of APP/PS1 transgenic mice.** Drugs were given twice weekly to mice at 4.5 month of age for 2 months, and the body weight was measured every two weeks. The data were expressed as mean ± SD. n = 8. Statistical analysis was performed using One-way ANOVA and Tukey’s test.

**
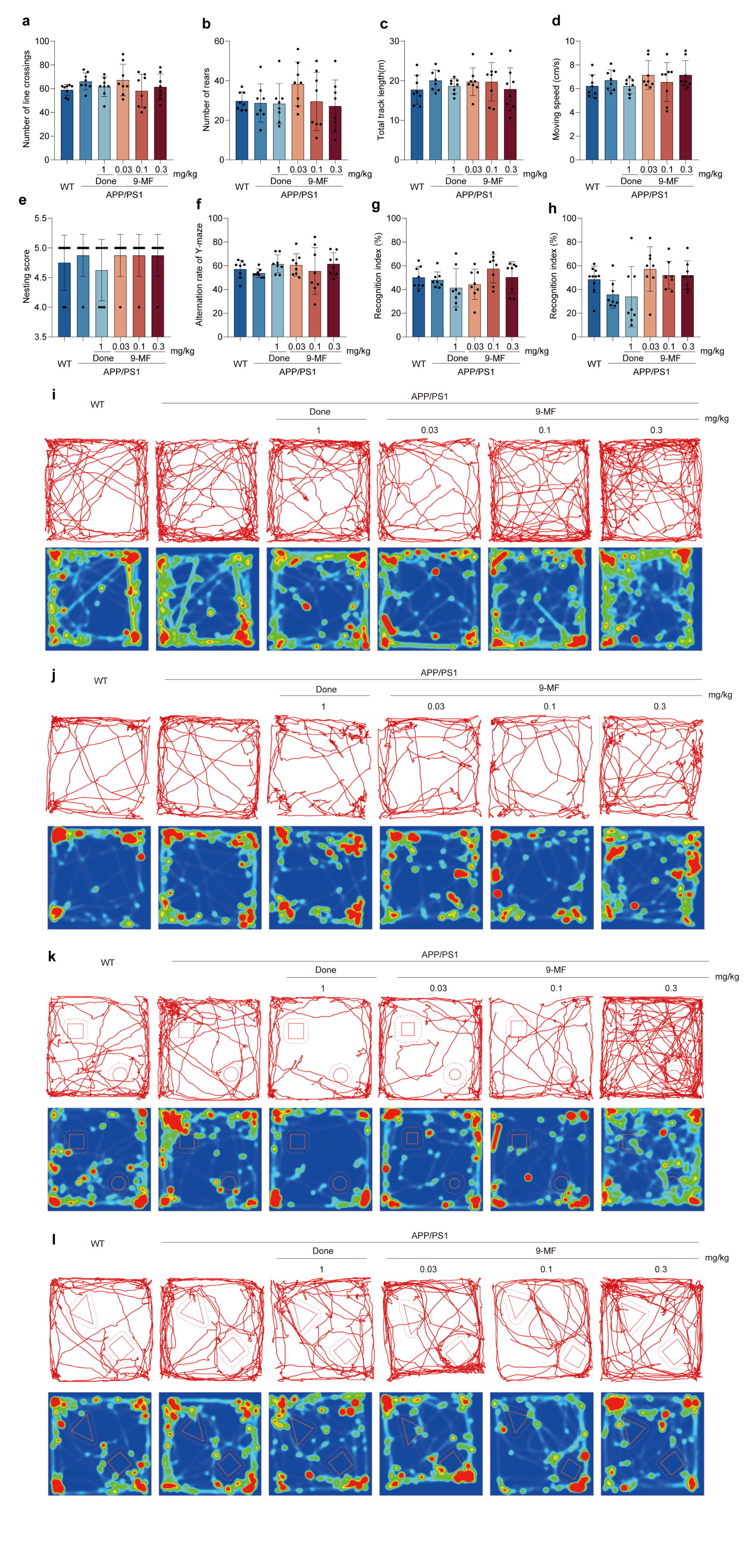
**

**Extended Data Figure 2 9-MF did not change the motor function and cognitive performance in APP/PS1 transgenic mice at 5-5.5 months of age.** In the open field tests, 9-MF did not significantly change (a) the number of crossings, (b) the number of rears, (c) total track length or (d) the moving speed in APP/PS1 transgenic mice at 5-5.5 months of age. (e) 9-MF did not significantly alter nesting scores in the nesting test or the time in closed arms in Y-maze tests in APP/PS1 transgenic mice at 5-5.5 months of age. 9-MF did not affect the recognition index during (g) the training session or (h) the retention session of NOR tests in APP/PS1 transgenic mice at 5-5.5 months of age. Amap and thermogram results of open field tests in APP/PS1 transgenic mice at (i) 5-5.5 and (j) 9-9.5 months of age, respectively. Representative heatmaps of trace for NOR tests in APP/PS1 transgenic mice at (k) 5-5.5 and (l) 9-9.5 months of age, receptively. The data were expressed as mean ± SD. n = 8. Statistical analysis was performed using One-way ANOVA and Tukey’s test.

**
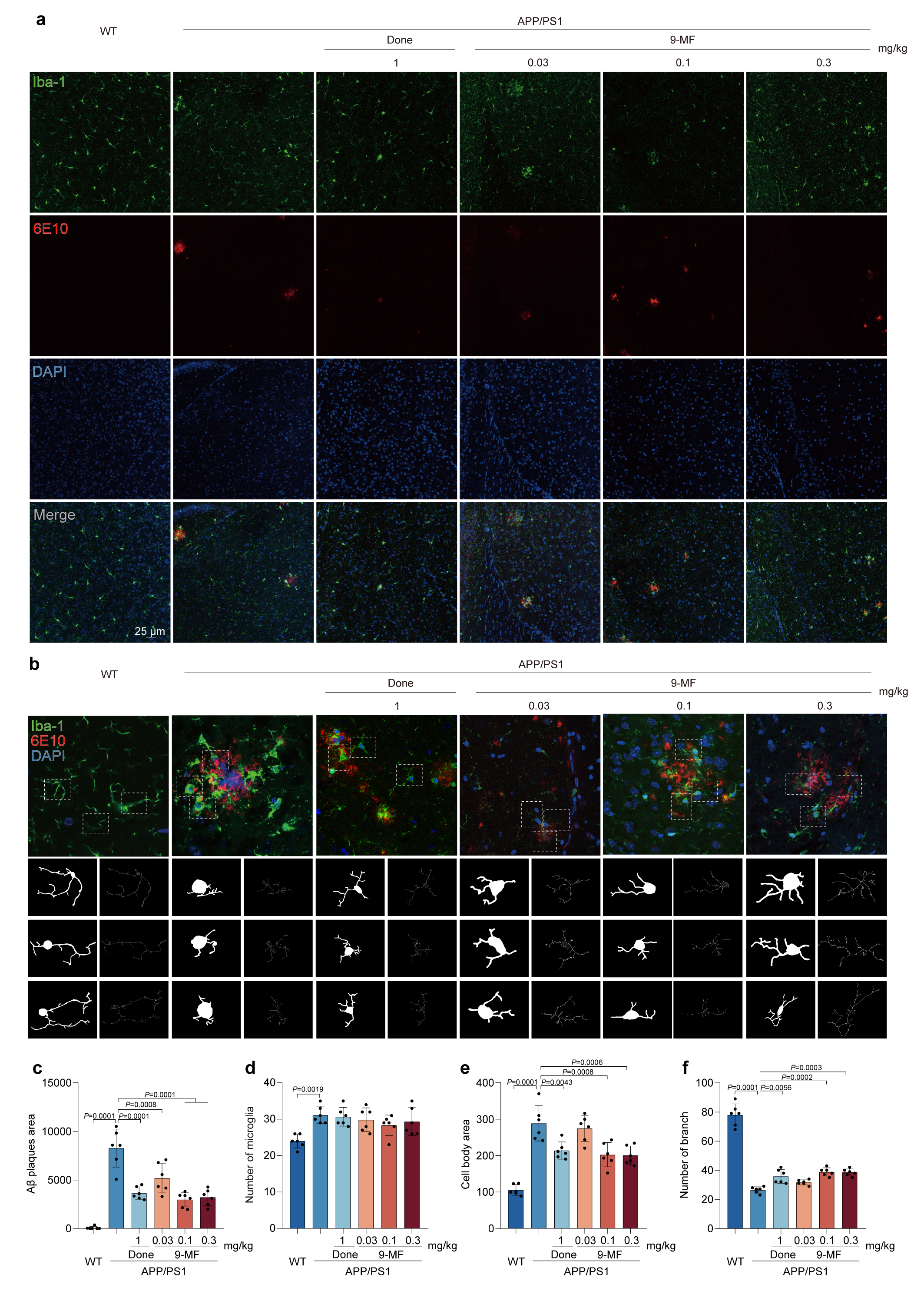
**

## Extended Data Figure 3 9-MF effectively reduces the amount of Aβ plaques and the overactivation of surrounding microglia in the cortex of APP/PS1 transgenic mice. (a) Representative immunofluorescent images of cortical slices stained with 6E10-positive plaques and Iba-1-positive cells (20[×](https://www.bing.com/ck/a?!&&p=fb33588e3f91a0b7JmltdHM9MTY5MTYyNTYwMCZpZ3VpZD0yYmQ0NjVlNy0zMzY5LTZkNjctMTdlMi03NzY5MzJiYjZjYzYmaW5zaWQ9NTQwMA&ptn=3&hsh=3&fclid=2bd465e7-3369-6d67-17e2-776932bb6cc6&psq=%e4%b9%98%e5%8f%b7&u=a1aHR0cHM6Ly9zeW1ibC5jYy9jbi8wMEQ3Lw&ntb=1) magnification). (b) Representative immunofluorescent images of microglia and Aβ co-localization and the cytoskeleton of microglia around Aβ plaques in the cortex (60[×](https://www.bing.com/ck/a?!&&p=fb33588e3f91a0b7JmltdHM9MTY5MTYyNTYwMCZpZ3VpZD0yYmQ0NjVlNy0zMzY5LTZkNjctMTdlMi03NzY5MzJiYjZjYzYmaW5zaWQ9NTQwMA&ptn=3&hsh=3&fclid=2bd465e7-3369-6d67-17e2-776932bb6cc6&psq=%e4%b9%98%e5%8f%b7&u=a1aHR0cHM6Ly9zeW1ibC5jYy9jbi8wMEQ3Lw&ntb=1) magnification). (c) The quantification of Aβ area in (a) was shown. (d) The number of microglia in (a) was shown. The quantification of (e) cell body area and (f) branches in (b) was shown. The data were expressed as mean ± SD. n = 6 in (c)-(f). Statistical analysis was performed using One-way ANOVA and Tukey’s test.


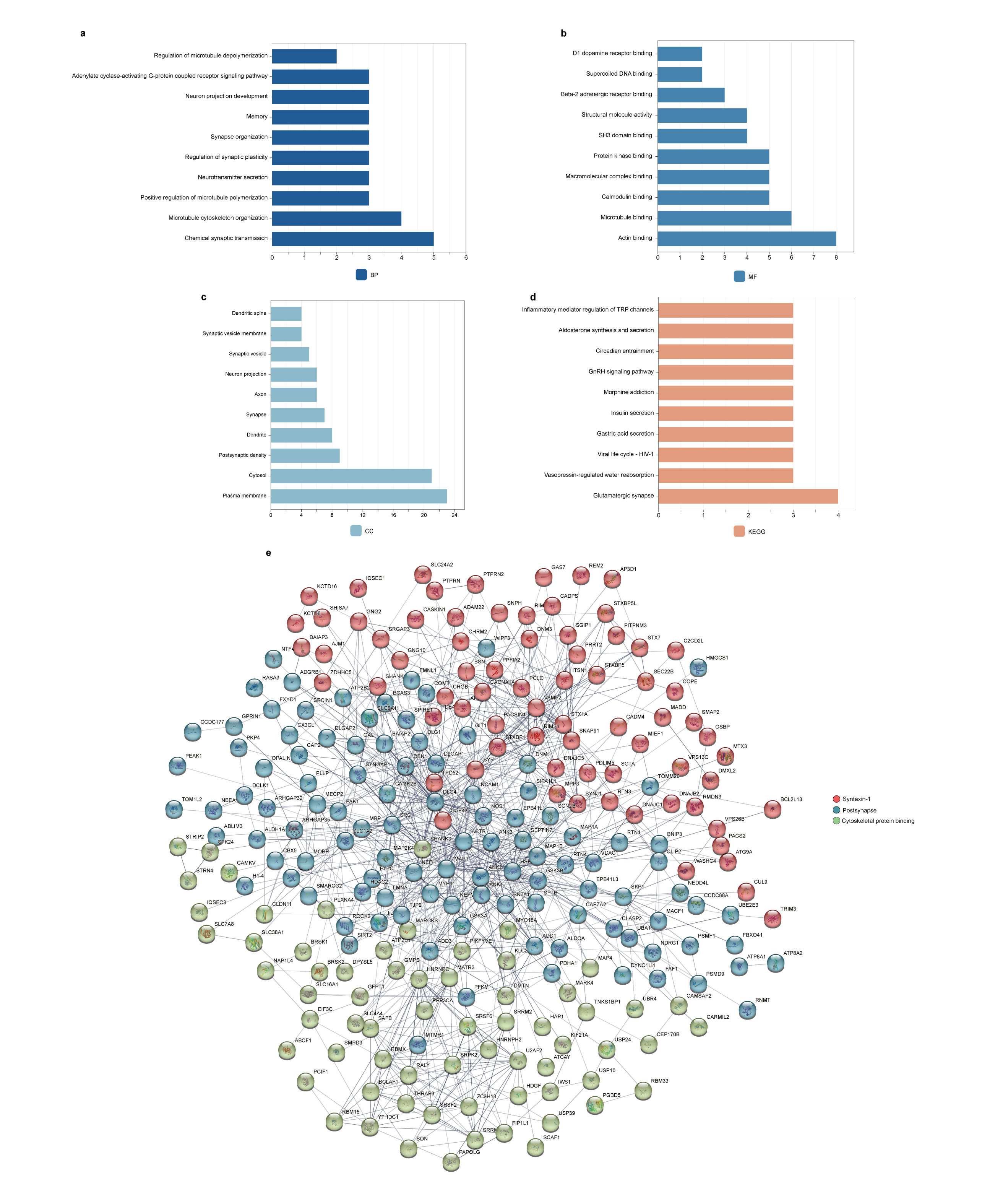


**Extended Data Figure 4 9-MF-regulated DPPs in APP/PS1 transgenic mice.** GO enrichment analysis of 9-MF-regulated DPPs in APP/PS1 transgenic mice. (a) BP: biological process; (b) MF: molecular function; (c) CC: cellular components. (d) KEGG analysis of 9-MF-regulated DPPs in APP/PS1 transgenic mice. x-axis indicates the count with the corresponding enriched pathway marked on y-axis. (e) A biological network was established from 9-MF-regulated DPPs in APP/PS1 transgenic mice. The interaction score was set as medium confidence (0.40, PPI enrichment p < 1 × 10^-16^). The strength of PPI is represented by the thickness of the middle line, and the nodes that are disconnected from the network are hidden. K-means algorithm is used to cluster the network.
